# Biomarkers of inflammation in infants with cystic fibrosis

**DOI:** 10.1186/s12931-017-0713-8

**Published:** 2018-01-08

**Authors:** Theresa A. Laguna, Cynthia B. Williams, Myra G. Nunez, Cole Welchlin-Bradford, Catherine E. Moen, Cavan S. Reilly, Chris H. Wendt

**Affiliations:** 1 Minnesota CF Center, Department of Pediatrics, University of Minnesota Masonic Children’s Hospital, 420 Delaware St. SE; MMC-742, Minneapolis, MN 55455 USA; 2 School of Public Health, Division of Biostatistics, University of Minnesota, Minneapolis, MN USA; 3 Department of Medicine, Division of Pulmonary, Allergy, Critical Care and Sleep Medicine, University of Minnesota and Veterans Administration Medical Center, Minneapolis, MN USA

**Keywords:** Inflammation, Lung function, Infection, Pediatrics, Bronchoalveolar lavage fluid

## Abstract

**Background:**

There are urgent needs for clinically relevant biomarkers to identify children with cystic fibrosis (CF) at risk for more progressive lung disease and to serve as outcome measures for clinical trials. Our objective was to investigate three targeted biomarkers in a population of asymptomatic CF infants.

**Methods:**

Urine, blood and lung function data were collected for 2 years from clinically stable infants diagnosed with CF by newborn screening. A subset of CF infants had bronchoscopy with lavage performed at 6 months and 1 year. Urine was collected quarterly from healthy control infants. Expectorated sputum and urine were collected quarterly for 2 years from clinically stable CF adults. Desmosine, club cell secretory protein (CCSP) and cathepsin B concentrations were measured and compared. Mixed effects models were used to identify associations between biomarker concentrations and clinical characteristics. Receiver operator characteristic curves were generated to investigate the sensitivity and specificity of the biomarkers.

**Results:**

Urinary cathepsin B was significantly higher in CF infants compared to healthy infants (*p* = 0.005). CF infant airway and urinary cathepsin B concentrations were significantly lower compared to adult CF subjects (*p* = 0.002 & *p* = 0.022, respectively). CF infant airway CCSP was significantly higher than adult CF subjects (*p* < 0.001). There was a significant correlation between CF infant plasma CCSP and BALF CCSP (*p* = 0.046). BALF CCSP was negatively associated with IL-8 (*p* = 0.017). There was no correlation between biomarker concentration and FEV_0.5_.

**Conclusions:**

Cathepsin B and CCSP show promise as biomarkers of inflammation in CF infants. Further study is needed.

**Electronic supplementary material:**

The online version of this article (10.1186/s12931-017-0713-8) contains supplementary material, which is available to authorized users.

## Background

Cystic fibrosis (CF) lung disease begins silently in infancy and is characterized by infection, chronic inflammation, bronchiectasis, progressive lung function decline and intermittent pulmonary exacerbations [1–5]. Early detection and treatment of pulmonary decline is key to optimal long-term outcome; however, physiologic measures of lung function and radiologic outcomes fail to capture the inflammation and infection that begins in infancy and is often silent in CF [6–8]. In addition, with the recent development of medications targeting the genetic mutations in CF, there is a need for outcome measures suitable for infants and young children for clinical trials. There are limited, non-invasive, objective tools available to help identify those CF infants at risk for a more rapidly progressive pulmonary course. This unmet need could be fulfilled by identifying biomarkers of lower airway inflammation and/or injury that are suitable both as “point-of-care” diagnostics and for monitoring longitudinal changes.

Neutrophilic inflammation, enzymatic protease activity and cytokine release are hallmarks of CF lung disease with the presence of neutrophil elastase (NE) in BALF known to be a risk factor for the development of bronchiectasis [9]. However, surveillance bronchoscopy with lavage is an invasive procedure that is not routinely performed in the United States, making the measurement of NE in young patients challenging. Biomarkers that reflect CF lung disease in easily obtained biological specimens are more feasible for application in both the clinical and research settings. Three biomarkers – desmosine, club cell secretory protein (CCSP) and cathepsin B have previously been associated with CF lung disease; however, their applicability to CF infants has not been investigated [10–18]. We have shown that sputum CCSP concentration was both higher in adults with CF in the outpatient setting compared to during a CF pulmonary exacerbation and negatively associated with sputum NE concentration [17]. CCSP is produced by bronchial epithelial cells and known to have anti-inflammatory properties and may reflect CF lung disease activity [17]. We have also shown that both sputum and urinary desmosine concentration decrease significantly during a CF pulmonary exacerbation [11, 12]. Previous work in premature infants and animals has suggested CCSP, cathepsin B and desmosine may all play a role in the development of chronic lung disease [19–22]. Desmosine is a breakdown product of elastin and is widely described as a non-specific biomarker of lung injury. Cathepsin B is an elastolytic cysteine protease that is linked to remodeling of extracellular matrix, bacterial infection and biofilm formation in the CF airways, specifically involving *Pseudomonas aeruginosa*, a known pathogen in CF [10, 13–16]*.* These three targeted biomarkers may provide an objective measure of the degree of inflammation or tissue destruction occurring in the lung, and if so, would be invaluable both for clinical management and as surrogate endpoints for clinical trials.

Our objective was to measure the concentration of desmosine, CCSP and cathepsin B during the first 2 years of life and correlate biomarker concentrations with markers of infection, inflammation and lung function. We also sought to compare biomarker concentrations measured in CF infants with those measured in a cohort of healthy infants and in an adult cohort of patients with CF with more established lung disease [17]. Some of the results of these studies have been previously reported in the form of an abstract [23].

## Methods

### Study Design for Infant Study

A single-center, two-year prospective cohort study of infants diagnosed with CF via newborn screening was performed. Infants were enrolled at their 3 month visit to the University of Minnesota (UMN) CF Clinic from 2009 to 2015. All infants with a confirmed diagnosis of CF [i.e. two known disease causing cystic fibrosis transmembrane conductance regulator (CFTR) mutations and/or a positive sweat chloride test by pilocarpine iontophoresis] were eligible to participate. Each subject had a blood sample taken and infant lung function testing (iPFT) performed every 6 months and provided a bag urine sample quarterly for 2 years. Under a separate study protocol, a subset of CF infants underwent a bronchoscopy with bronchoalveolar lavage following their iPFT at 6 months of age and 1 year. We collected demographic data and performed an upper airway nasopharyngeal (NP) culture quarterly at clinic visits. Healthy, control infants recruited from a local pediatric clinic provided only a bag urine sample quarterly for 2 years. Infants were recruited at outpatient well-child visits and were required to have no history of prematurity or known lung disease to participate. The UMN Institutional Review Board (IRB) approved study protocols and written informed consent was obtained from each of the subject’s parents or legal guardians.

### Study design for adult CF study

A single-center, two-year prospective cohort study of patients with CF during times of pulmonary exacerbation (hospitalization) and times of clinical stability (outpatient clinic visits) was performed. Subjects with a confirmed diagnosis of CF were enrolled upon admission for treatment of a pulmonary exacerbation from 2008 to 2011 [24]. For study purposes, a pulmonary exacerbation was defined as the need for hospitalization for intravenous (IV) antibiotics. Each subject provided an expectorated sputum and urine sample at up to three time points. Upon discharge, expectorated sputum and urine samples were collected quarterly at outpatient visits for 2 years. For the purposes of this study, only specimens collected during outpatient visits were analyzed. If a CF patient was on IV antibiotics or received a diagnosis of a CF pulmonary exacerbation at a clinic visit, samples were excluded from the analysis. The study protocol was approved by the UMN IRB and informed consent and/or assent were obtained from each of the subjects and/or their parents or guardians.

### Procedures

#### Infant lung function testing

Using the Infant Plethysmograph (IPL) from nSpire Health, this procedure is attempted on CF infants at the Minnesota CF Center every 6 months for the first 2 years of life as standard-of-care. For the iPFT procedure, infants were sedated with oral chloral hydrate and oral hydroxyzine. We were unable to obtain iPFT data on the infants that were unable to sleep through the entire procedure. The forced expiratory volume in 0.5 s (FEV_0.5_) by the raised volume rapid thoracoabdominal compression technique (RVRTC) was collected [25–27].

#### BALF specimen collection/processing

As part of a separate, IRB-approved study protocol, bronchoscopy with lavage was performed on a small number of CF infants immediately following the iPFT procedure to take advantage of the already administered sedation. Two aliquots of 1 mL/kg of sterile normal saline were instilled into the right middle lobe. All BALF was pooled and immediately placed on ice. One aliquot was sent to the UMN clinical laboratory for cell count and differential and the qualitative identification of respiratory pathogens by standard microbiological techniques [28–30]. The UMN Microbiology Laboratory does not quantify the amount of bacteria present on standard culture. The remaining BALF was centrifuged at 250×g for 10 min at 4 °C. The supernatant was transferred with a sterile pipette to a separate tube, protease inhibitors were added, and the mixture centrifuged at 4000×g for 20 min at 4 °C. The sample was stored at −80 °C for future analysis. CCSP and cathepsin B concentration were measured in BALF as described below.

#### NP specimen collection

Nasopharyngeal specimens collected by inserting a sterile suction catheter through the nose followed by gentle suction are standard of care at the Pediatric UMN CF Center. The specimen was sent for the same qualitative identification of respiratory pathogens as per the BALF specimen.

#### Sputum collection/processing

Expectorated sputum from adults with CF was processed as previously described and frozen immediately after collection at −80 °C prior to analysis [31]. CCSP and cathepsin B concentrations were measured in sputum as described below.

#### Urine collection/processing

A bag urine sample was collected from all infants and a clean catch specimen was collected from adult CF subjects. Urine was centrifuged at 4000RPM for 10 min and the supernatant was aliquoted and stored at −80 °C for future analysis. Urine samples were shipped on dry ice for desmosine analysis. Cathepsin B concentration was also measured in urine as described below.

#### Blood collection/processing

A blood sample was collected from all CF subjects in standard BD vacutainer tubes and centrifuged at 4000 RPM for 10 min. The resultant plasma and serum supernatants were aliquoted and stored at −80 °C for future analysis.

### Laboratory assays

#### Biomarker assays

CCSP concentration was measured in plasma, sputum and BALF supernatant treated with protease inhibitors using a human-specific competitive ELISA assay (Clarassance, Inc.; Rockville, MD, USA). The limit of detection (LOD) for this assay was 5 ng/mL. Four parameter logistic curves were used that ranged from 5 to 500 ng/mL. The intra-assay coefficient of variation (CV) was <12% and the inter-assay CV was <10%. Specimens were run in duplicate and a human serum control was used to ensure reproducibility. Desmosine concentration was measured in infant urine using a liquid chromatography, mass spectrometry-based assay at the University of Dundee [32]. Cathepsin B concentration was measured in BALF and sputum supernatant treated with protease inhibitors and in urine using a competitive ELISA assay (R&D Systems, Inc.; Minneapolis, MN, USA). The reported mean minimum detectable concentration for this assay was 0.016 ng/mL. Four parameter logistic curves were used that ranged from 0 to 10 ng/mL. The intra-assay CV was <10% and the inter-assay CV was <10%.

#### BALF analysis

BALF NE activity was quantified by a spectrophotometric assay based on the hydrolysis of the specific substrate MeO-suc-Ala-Ala-Pro-Ala-p-nitroanilide (Sigma Chemical Co.; St. Louis, MO). IL-8 was measured as a component of an eight-plex cytokine assay (EMD Millipore Corporation; Billerica, MA).

#### Urine analysis

Urinary creatinine and specific gravity were measured for normalization purposes [33].

#### Statistical analysis

Descriptive statistics include the mean and standard deviation or the median and range, where specified. Linear mixed effects models were used to model the logarithm of targeted biomarker levels that were longitudinally assessed with subject-specific, additive random effects. Analysis of urine samples controlled for creatinine and specific gravity by taking the log and including them as covariates. Sputum and BALF biomarker concentrations were not normalized prior to analysis. To test for an association between urinary cathepsin B levels and CF status, a model with the log of cathepsin B as the response variable and the log of specific gravity, the log of creatinine, age, CF status and the interaction between age and the indicator for CF status was fit. The test for an association between BALF CCSP and plasma CCSP was based on a model which had BALF CCSP as a response variable and included no other covariates (although the results were not sensitive to inclusion of gender and age). A similar analysis detected the association between CCSP and IL-8. Two sample t-tests were used to test for differences in biomarker concentration between infants and older subjects with CF [17]. Receiver operator characteristic (ROC) curves were generated to examine the sensitivity and specificity of the biomarkers. All calculations were conducted using R version 2.15.2 and mixed effects models were fit using the lme function in the nlme package [34].

## Results

### Study population

Demographic data for the study population is presented in Table 1. The median FEV_0.5_% predicted (range) for those infants who received an iPFT (*n* = 21) was 96% predicted (range, 66–125%). The predominant genotype of the infant CF population was F508del/F508del. Eleven of the 12 infants with the genotype F508del/other were pancreatic insufficient as confirmed by a pancreatic stool elastase <200 mcg/g of stool. The adult CF cohort had a median age of 29.1 years, a median FEV_1_ of 56% predicted at baseline and all were confirmed pancreatic insufficient.

**Table 1:** Demographics of CF Infant Cohort, CF infants who received a bronchoscopy, Healthy Control Infants and Adult CF Subjects

	Characteristic	Value
CF Infants (Total)	Subjects	33
	Female:Male	20:13
	Age at enrollment (months)	3.5 (0.4–13.0)
	Initial FEV_0.5_ (% predicted)	96 (66–125%)
	# urine samples collected	130
	# blood samples collected	41
	# iPFT procedures performed	36
	*P. aeruginosa* positive during study	5
	F508del/F508del	19 (58%)
	F508del/other	12 (36%)
	other/other	2 (6%)
CF Infants (BALF)	Subjects	8
	Female:Male	6:2
	BALF Samples	12
	Age at first BAL (months)	6.1 (5.7–6.5)
	Age at second BAL (months)	12.2 (11.8–13.2)
	FEV0.5 at first BAL (% predicted)	98 (69–125%)
	F508del/F508del	7 (88%)
	F508del/G551D	1 (12%)
Healthy Infants	Subjects	40
	Female:Male	20:20
	Age at enrollment (months)	2.4 (1.8–12.0)
	# urine samples collected	90
CF Adults	Subjects	36
	Female:Male	19:17
	Age at enrollment (years)	29.8 (13.0–56.6)
	FEV1 (% predicted), clinic	56 (18–96%)
	# urine samples collected	125
	# sputum samples collected	109
	# Clinic Visits/subject	5 (1–8)
	F508del/F508del	20 (56%)
	F508del/other	14 (39%)
	other/other	2 (5%)

Values are presented as number (%) or median (range)

### CCSP, Desmosine and Cathepsin B concentrations in infants

**Fig. 1:**
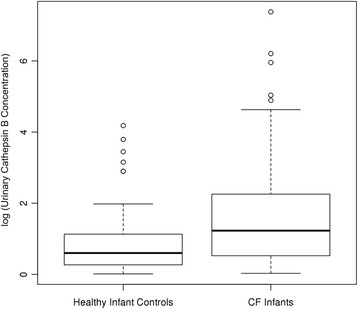
Urinary cathepsin B concentration is higher in CF infants compared to healthy control infants (*p* = 0.005)

**Fig. 2:**
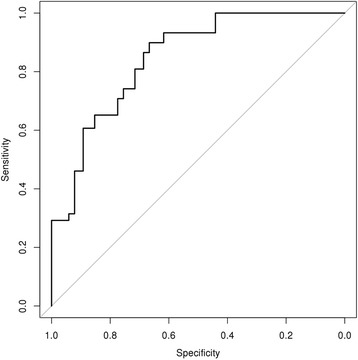
ROC curve for the ability of urinary cathepsin B concentration to distinguish between CF and healthy, control infants

**Fig. 3:**
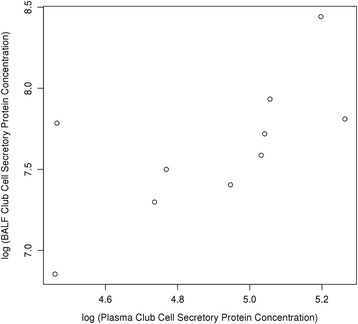
A positive, significant association between CF infant plasma CCSP and concentration and CF infants BALF concentration was observed (*p* = 0.046)

BALF CCSP concentration, plasma CCSP concentration and urinary desmosine concentration did not change significantly in CF infants during the 2 year study period (Additional file 1 shows cumulative biomarker concentrations). Urinary cathepsin B concentration was higher in CF infants than in healthy controls (*p* = 0.005, Fig. 1). The estimated ROC curve had an area under the curve of 0.85 (95% CI: 0.79, 0.90) using urinary cathepsin B concentration to distinguish between CF and healthy infants (Fig. 2). There was a positive, significant association between CF infant plasma CCSP concentration with CF infant BALF CCSP concentration (Fig. 3**,** *p* = 0.046). There was not a statistically significant association between CF urinary and BALF cathepsin B concentration. A positive, statistically significant association was detected between CF infant urinary cathepsin B concentration and urinary desmosine concentration (*p* = 0.006; Additional file 2).

### Comparison of CF infant biomarker concentrations with adult CF cohort

**Fig. 4:**
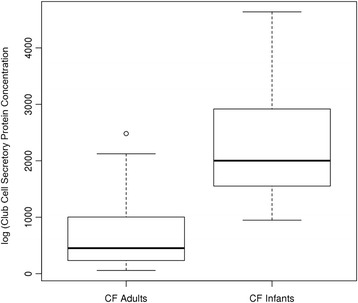
CF infants had a significantly higher CCSP concentration measured in BALF compared to that in adult CF sputum (*p* = 3.868e^-0.6^)

**Fig. 5:**
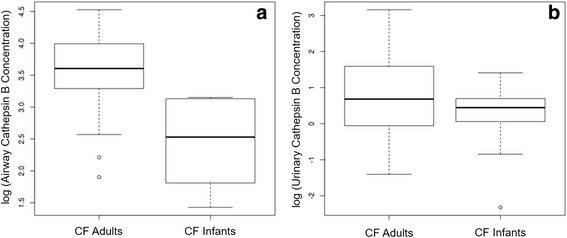
**a** CF infant BALF cathepsin B concentration was significant lower than that in adult sputum (*p* = 0.002), and (**b**) CF infant urinary cathepsin B concentration was significantly lower than that in adult urine (*p* = 0.022)

CF infants had a BALF CCSP concentration that was significantly higher than that in adult CF sputum collected during an outpatient clinic visit (*p* < 0.001, Fig. 4) [17]. CF infant BALF and urinary cathepsin B concentration was significantly lower than adult sputum and urinary cathepsin B concentration collected during an outpatient clinic visit (*p* = 0.002 and *p* = 0.022, respectively, Fig. 5).

### Correlations of targeted biomarker concentrations with lung function and markers of infection and inflammation in CF infants

There was no significant association between % neutrophils or absolute neutrophil count in BALF, BALF NE and CCSP, Cathepsin B or desmosine concentrations (Additional file 3). There was a positive, significant association between plasma CCSP concentration and the presence of *M. catarrhalis* (*p* = 0.012), *S. pneumoniae* (*p* = 0.037) and *S. aureus* (*p* = 0.020) in BALF culture. Additional file 4 shows the number of CF infants who were positive for each of the CF pathogens on standard culture. There was no association detected between targeted biomarker concentration and the presence of *P. aeruginosa*. CF infant BALF CCSP was negatively associated with BALF IL-8 concentration (*p* = 0.017). There was no correlation between CF infant BALF biomarkers and the additional cytokines measured; however, cytokine concentrations in infant BALF are represented in Additional file 5. There was no correlation between targeted biomarker concentration and FEV_0.5_ (Additional file 6 shows the analysis of FEV_0.5_ and the targeted biomarkers).

## Discussion

There is an unmet need in this population, with no objective means by which to identify and follow the progression of lung disease in CF infants. Through the comparison to healthy controls and older subjects with CF, our findings suggest that CCSP and cathepsin B are potential biomarkers of lung disease activity in infants and should be investigated in future longitudinal and mechanistic studies.

The search for biomarkers of CF lung disease in infants and children remains challenging as they do not typically expectorate sputum, traditional lung function testing is not attainable, BALF is not routinely obtained, cumulative radiation exposure is a concern and phlebotomy can be difficult [35–38]. In addition to our prior work establishing a relationship between CCSP and desmosine concentration to CF pulmonary exacerbation in older CF patients, additional work in bronchial secretions in cell culture led us to investigate these three targeted biomarkers in a CF infant cohort [11, 12, 17, 18]. Utilizing quantitative proteomics to investigate the in vitro bronchial epithelial secretome, uteroglobin-related protein 2 (i.e. CCSP) was found to be decreased and cathepsin B was increased in CF secretions compared to non-CF secretions [18]. These findings were in the absence of inflammatory cells and pathogens, suggesting altered immune and injury repair proteins related to CFTR dysfunction [18]. These three biomarkers have shown clinical and biological relevance in CF, providing a foundation for this investigation in infants.

Although we did not find that CCSP or desmosine concentration changed significantly over the first 2 years of life, we identified differences in cathepsin B concentration between our CF infants, healthy controls and older subjects with CF. We are the first to report the presence of cathepsin B in the urine and BALF of infants with CF. Although previous work has identified the presence of NE in CF infant BALF, ours is the first to document the presence of a cysteine protease in the CF infant airway [9]. Cathepsin B concentration was found to be increased in BALF from baboons who subsequently developed BPD, suggesting a role in the development of chronic lung disease [21]. CF infants had a significantly higher urinary cathepsin B concentration than normal, healthy infants without CF. Lung disease in CF is known to have its onset early in life and the higher concentration of cathepsin B is a potential indication of early airway damage. In addition, the CF infants had significantly lower concentrations of cathepsin B in their urine and BALF compared to an older cohort of subjects with CF who had more established lung disease. Only 5 of the 33 infants with CF in our study were infected with *P. aeruginosa*, and these subjects did not have a higher concentration of urinary or BALF cathepsin B, confirming a previous finding that cathepsins are not reliable markers of *P. aeruginosa* infection in CF [39]. Urinary cathepsin B concentration was positively associated with urinary desmosine concentration, confirming the presence of these two deleterious proteins in CF infants. An elevated urinary desmosine concentration in premature infants has been suggested as a biochemical marker of damage to the developing lung, and also has been shown to decrease during inpatient treatment for a CF pulmonary exacerbation in older patients [12, 22, 40]. Cathepsin B in the airway of subjects with CF is thought to be a marker of inflammation; however, our study was not designed to investigate mechanism of action.

CCSP concentration was significantly increased in infant BALF compared to our older subjects with CF. In addition, BALF CCSP concentration was negatively associated with IL-8 and positively correlated with blood CCSP concentration. Our previous longitudinal studies of sputum CCSP in CF have shown increased concentrations during times of stability compared to hospitalization for a CF pulmonary exacerbation [17]. CCSP has anti-inflammatory properties and is primarily produced by non-ciliated Club Cells in the conducting airways. Club cells secrete CCSP in very high concentrations in the epithelial lining fluid where it modulates the production and activity of phospholipase A_2_, interferon-γ and tumor necrosis factor-α, serving to protect the lung against oxidative stress [41–43]. Given that infants still have relatively healthy airways compared to adults with CF, they may have a higher density of intact Club Cells in the airways, leading to a higher measureable concentration of CCSP. Interestingly, CCSP has been studied in premature infants in the context of the development of bronchopulmonary dysplasia (BPD). CCSP concentration in tracheal aspirates from premature infants increases with age and in response to infection [19, 44] . Airway CCSP concentration was lower and not inducible in those premature infants who developed BPD or died, compared to those infants who did not develop BPD [20]. Although we do not have airway CCSP concentration in healthy control infants for comparison, infants with CF may have a surge of CCSP during the first year of life as an anti-inflammatory, anti-infectious response to the development of CF lung disease. The positive correlation between BALF and plasma CCSP concentration suggests that plasma levels may be a surrogate for airway CCSP concentration. Plasma CCSP concentration was also positively associated with bacteria not thought to be pathogenic in CF. Although the mechanistic role of CCSP in the development of CF lung disease is unclear, our findings suggest CF infants have significantly more of this biomarker detected in their airways and this was associated with less airway inflammation.

Our study is not without limitation. Our cohort of CF infants was small, and the subset who received a bronchoscopy with lavage had even more limited numbers. We compared biomarker concentration in two airway specimens (BALF and expectorated sputum) and in two CF populations (adults and children). Comparing the concentration of cellular and non-cellular components in BALF with sputum introduces a dilution factor; however, “normalization” of BALF for epithelial lining fluid (ELF) is not recommended due to a lack of a reliable method for its quantification [45]. Previous work in CF has determined that sputum may be a sample more highly concentrated in inflammatory mediators than BALF [46]. In our study, the CCSP concentration in BALF was significantly higher than sputum, and the difference in cathepsin B concentration between BALF and sputum was strengthened by a similar difference in urinary cathepsin B concentration. Although comparing an older CF cohort with CF infants may seem unorthodox, other validated biomarkers of CF lung disease reflect disease activity, regardless of age. Both sputum NE and IL-8 concentrations are negatively associated with lung function and are correlates of disease severity [36, 47]. We anticipate that CCSP and cathepsin B concentrations may behave in a similar fashion. This study was not designed to elucidate the mechanisms of action of the three targeted biomarkers; instead, we identified differences in cohorts of CF and healthy subjects that can serve to generate hypotheses for further longitudinal and mechanistic studies. Finally, we measured cathepsin B using an ELISA and did not measure protease activity.

## Conclusions

Our study is the first to measure CCSP and cathepsin B in infants with CF and compare these concentrations with those in control infants and older subjects with CF. We have identified CCSP and cathepsin B as potential biomarkers of CF lung disease and future studies are needed to better elucidate the mechanisms underlying their presence.

## Additional files

Additional file 1: Cumulative data for targeted biomarkers in healthy control and CF infants. (PDF 49 kb)

Additional file 2: Plot of urinary cathepsin B concentration vs. urinary desmosine concentration. (JPEG 157 kb)

Additional file 3: Additional plots of inflammatory marker analysis. (JPEG 167 kb)

Additional file 4: Table summarizing the culture results for all CF infants. (PDF 34 kb)

Additional file 5: Additional plots of cytokine concentrations in CF infant BALF. (JPEG 134 kb)

Additional file 6: Plots summarizing the statistical analysis of FEV_0.5_ with targeted biomarkers. (JPEG 159 kb)

## Abbreviation

BALF: Bronchoalveolar lavage fluid
BPD: Bronchopulmonary dysplasia
CCSP: Club cell secretory protein
CF: Cystic fibrosis
CFTR: Cystic fibrosis transmembrane conductance regulator
CV: Coefficient of variation
FEV_0.5_: Forced expiratory volume in 0.5 s
FEV_1_: Forced expiratory volume in 1 s
FVC: Forced vital capacity
HRCT: High-resolution computed tomography
IL-8: Interleukin-8
iPFT: Infant pulmonary function testing
IRB: Institutional review board
LCI: Lung clearance index
LOD: Limit of detection
NBS: Newborn screening
NE: Neutrophil elastase
NP: Nasopharyngeal
ROC: Receiver operator characteristic
RVRTC: Raised volume rapid thoracoabdominal technique
UMN: University of Minnesota
