# Supplementary material for: Biomarkers of inflammation in infants with cystic fibrosis
Source: Respir Res. 2018 Jan 8;19:6. doi: 10.1186/s12931-017-0713-8 (PMC5759377; doi:10.1186/s12931-017-0713-8)
Supplement: Supplementary file 1 — Cumulative data for targeted biomarkers in healthy control and CF infants. (PDF 49 kb) [file 12931_2017_713_MOESM1_ESM.pdf]

	Healthy	CF
CCSP: BAL (ng/mL)	NA (NA, NA)	150.35 (109.08, 164.47)
CCSP: plasma (ng/mL)	NA (NA, NA)	2111.25 (1767.34, 2500.56)
Desmosine: urine (pmD/ml)	97 (7, 962.77)	279.07 (216.08, 467.79)
Desmosine: urine (pmD/mgCr)	30.25 (23.81, 67.62)	62.25 (40.5, 230.81)
Desmosine: BAL (pmD/ml)	NA (NA, NA)	10.29 (4.91, 31.16)
Desmosine: BAL (pmD/mgP)	NA (NA, NA)	269.48 (82.5, 518.04)
Cathepsin B: urine (ng/mL)	0.59 (0.27, 0.91)	0.83 (0.4, 2.46)
Cathepsin B: BAL (ng/mL)	NA (NA, NA)	10.79 (6.79, 14.52)

Additional File #1

Table 1: Summary of biomarkers in healthy controls and infants with cystic fibrosis at the earliest study visit with near complete data. Data are summarized in terms of medians and quartiles.
