# Supplementary figures and images for: Biomarkers of inflammation in infants with cystic fibrosis
Source: Respir Res. 2018 Jan 8;19:6. doi: 10.1186/s12931-017-0713-8 (PMC5759377; doi:10.1186/s12931-017-0713-8)

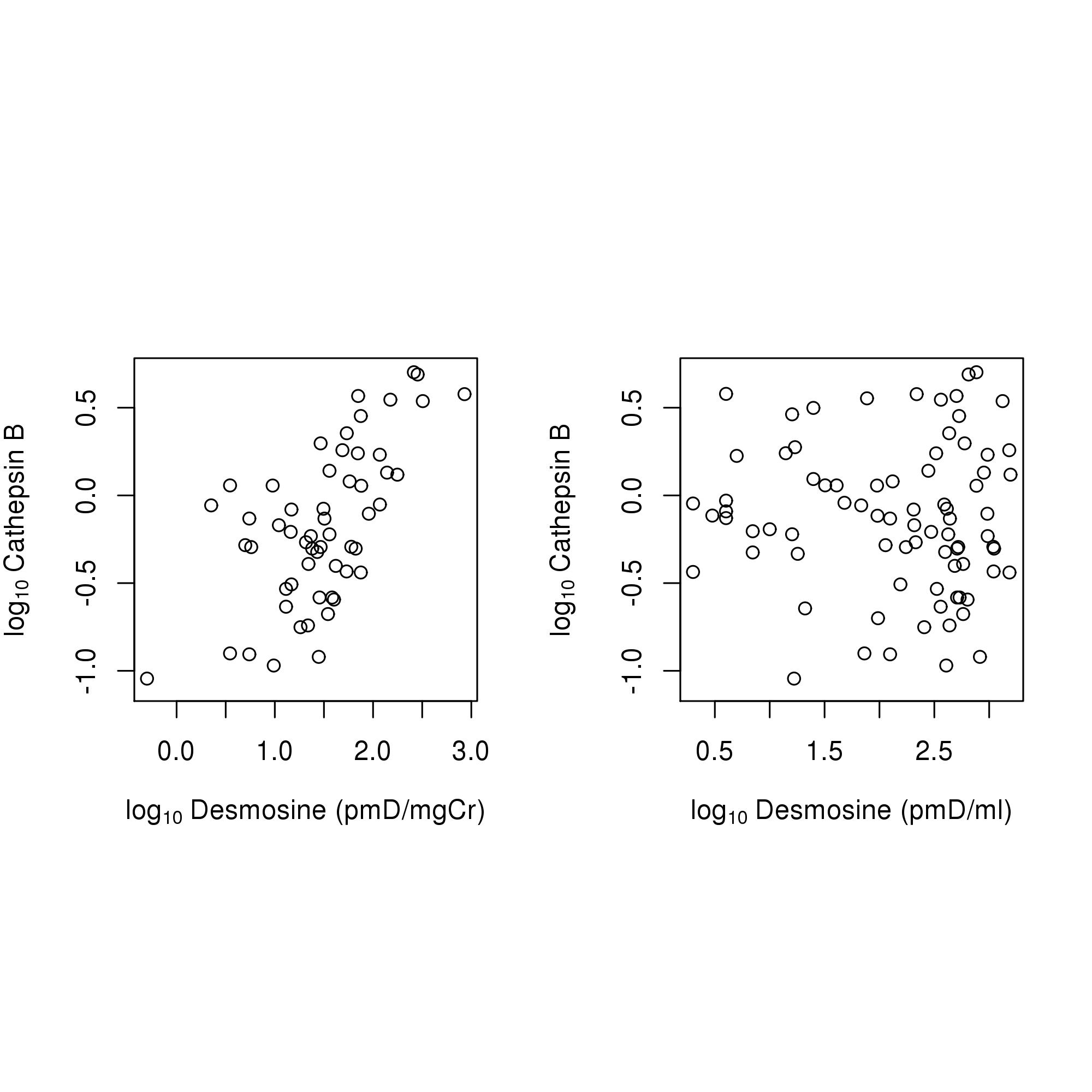

Supplement: Supplementary file 2 — Plot of urinary cathepsin B concentration vs. urinary desmosine concentration. (JPEG 157 kb) [file 12931_2017_713_MOESM2_ESM.jpg]

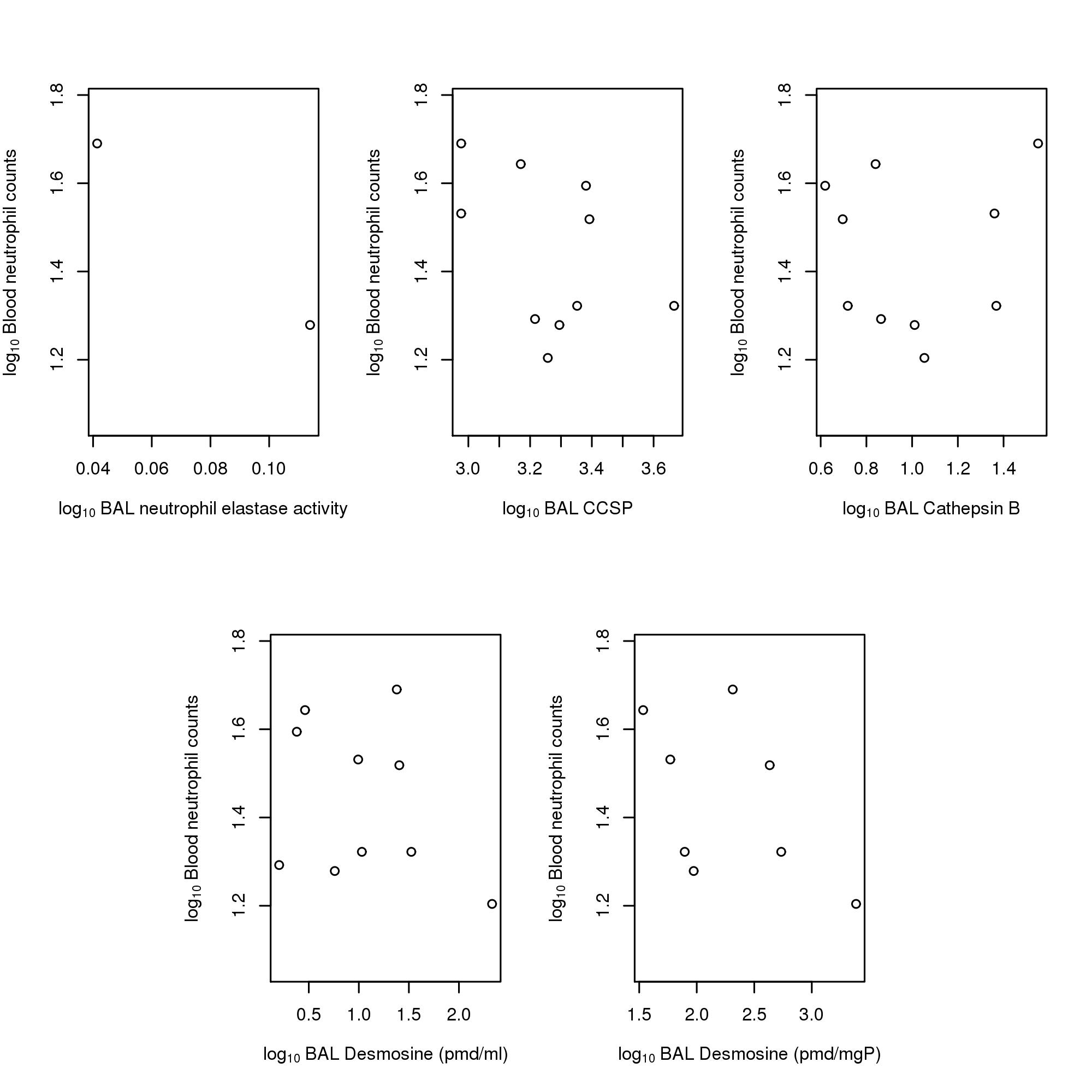

Supplement: Supplementary file 3 — Additional plots of inflammatory marker analysis. (JPEG 167 kb) [file 12931_2017_713_MOESM3_ESM.jpg]

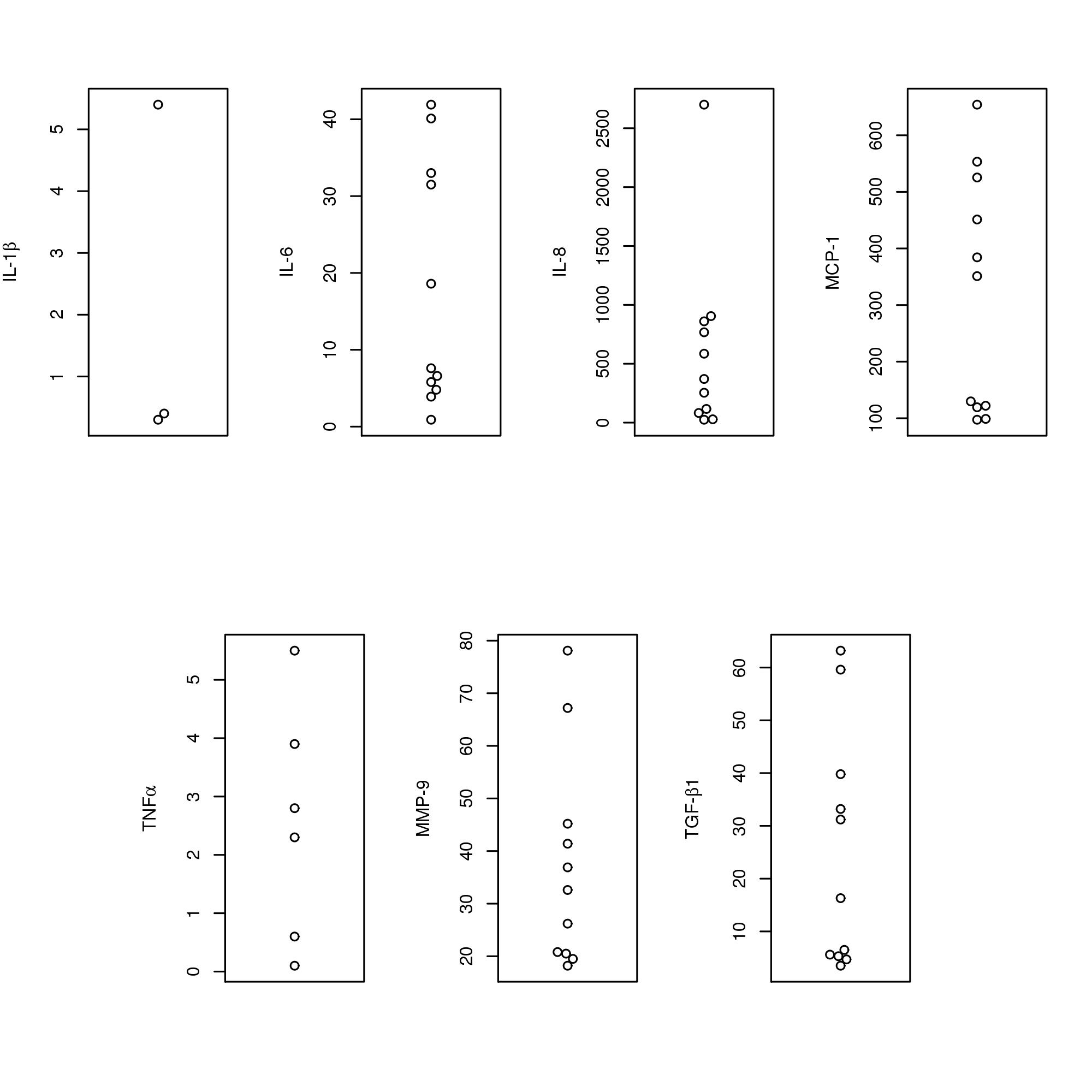

Supplement: Supplementary file 5 — Additional plots of cytokine concentrations in CF infant BALF. (JPEG 134 kb) [file 12931_2017_713_MOESM5_ESM.jpg]

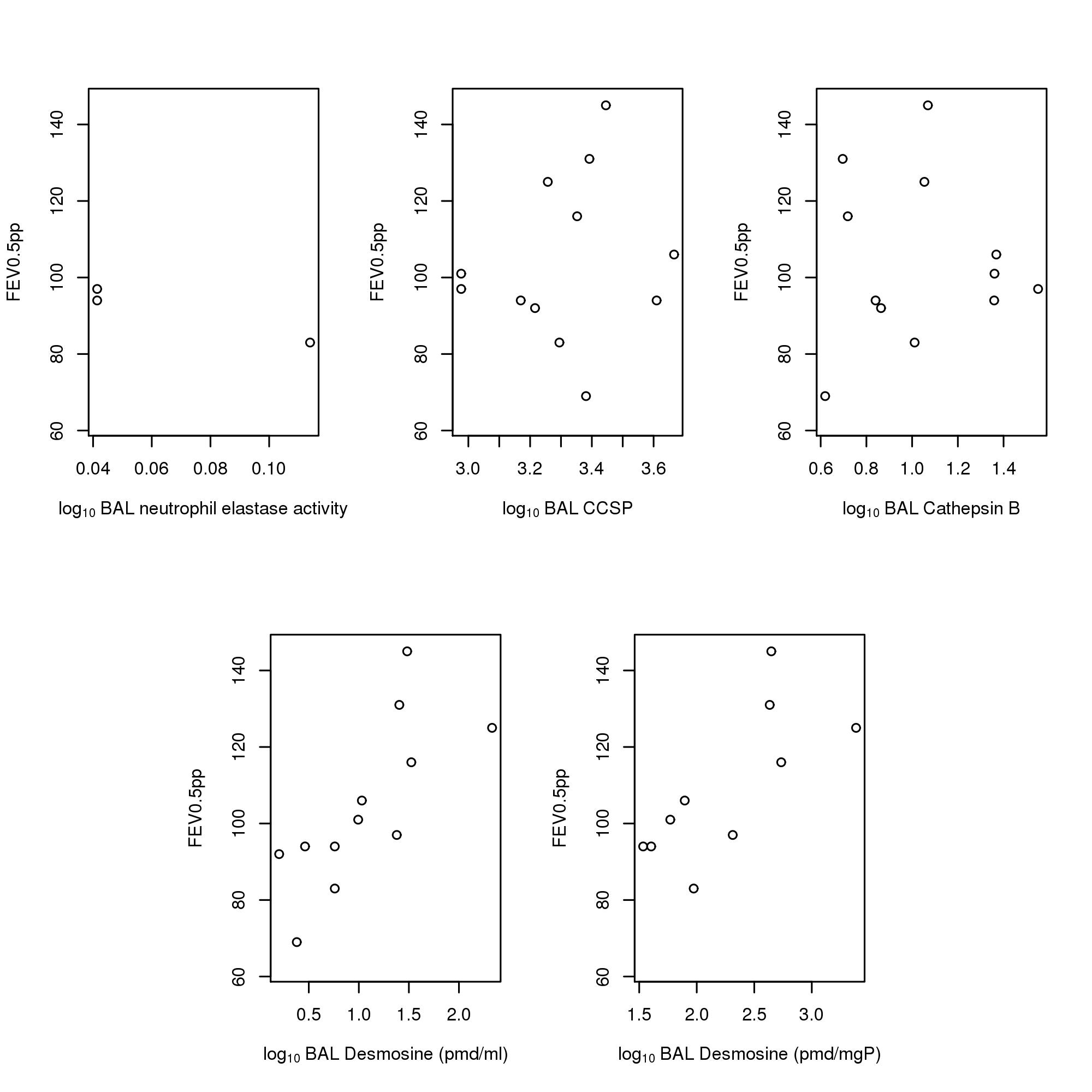

Supplement: Supplementary file 6 — Plots summarizing the statistical analysis of FEV0.5 with targeted biomarkers. (JPEG 159 kb) [file 12931_2017_713_MOESM6_ESM.jpg]
