# Supplementary material for: Biomarkers of inflammation in infants with cystic fibrosis
Source: Respir Res. 2018 Jan 8;19:6. doi: 10.1186/s12931-017-0713-8 (PMC5759377; doi:10.1186/s12931-017-0713-8)
Supplement: Supplementary file 4 — Table summarizing the culture results for all CF infants. (PDF 34 kb) [file 12931_2017_713_MOESM4_ESM.pdf]

	Number Negative	Number Positive
Acinetobacter baumannii complex	99	1
Acinetobacter lwoffi	99	1
Acinetobacter species	99	1
Bacillus cereus group, not anthracis	99	1
Beta hemolytic Streptococcus group A	97	3
Beta hemolytic Streptococcus, not group A	94	6
Bipolaris Species	99	1
Coagulase Positive Staphylococcus	100	0
Enterobacter cloacae complex	96	4
Escherichia coli	91	9
Escherichia hermannii	99	1
Haemophilus influenzae	78	22
Haemophilus parainfluenzae	99	1
Klebsiella oxytoca	98	2
Klebsiella pneumoniae	96	4
Methicillin resistant Staphylococcus aureus (MRSA)	98	2
Moraxella (Branhamella) catarrhalis	91	9
Pseudomonas aeruginosa	95	5
Proteus mirabilis	99	1
Paracoccus yeeii	99	1
Pantoea species	99	1
Staphylococcus aureus	53	47
Staphylococcus intermedius	99	1
Stenotrophomonas maltophilia	98	2
Serratia marcescens	99	1
Streptococcus pneumoniae	88	12
Staphylococcus pseudointermedius	99	1

Additional File #4

Table 2: The numbers of samples from cystic fibrosis infants that test positive for a collection of microorganisms.
